# Synchronous Double Primary Hepatocellular Carcinoma and Intrahepatic Cholangiocarcinoma: A Case Report

**DOI:** 10.70352/scrj.cr.25-0720

**Published:** 2026-04-15

**Authors:** Takahiro Yoshimura, Hironori Hayashi, Kazuki Kato, Shinichi Nakanuma, Yuichiro Furutani, Daisuke Fujimori, Hiroki Kitabayashi, Koichiro Sawada, Masanori Kotake, Kaeko Oyama, Takuo Hara, Shintaro Yagi

**Affiliations:** 1 Department of Surgery, Kouseiren Takaoka Hospital, Takaoka, Toyama, Japan; 2 Department of Hepato-biliary-pancreatic and Transplant Surgery, Kanazawa University Hospital, Kanazawa, Ishikawa, Japan

**Keywords:** chronic liver disease, double primary hepatic cancer, hepatectomy, hepatocellular carcinoma, intrahepatic cholangiocarcinoma

## Abstract

**INTRODUCTION:**

Hepatocellular carcinoma (HCC) and intrahepatic cholangiocarcinoma (iCCA) are the most common and second most common primary liver cancers, respectively. In contrast, double primary hepatic cancer (DPHC), the synchronous occurrence of these two tumors in different locations of the liver, is an uncommon pathology, occurring in less than 0.8% of all liver malignancies.

**CASE PRESENTATION:**

A man in his 70s was referred to our department for the treatment of multiple liver tumors. He had previously been treated with a direct-acting antiviral agent for chronic liver disease due to hepatitis C virus infection and alcohol consumption. His carbohydrate antigen 19-9 (CA19-9) and des-γ-carboxy prothrombin levels were 36058 U/mL and 159 mAU/mL, respectively. Imaging studies revealed two separate lesions with different contrast features in segments VIII (SVIII, 2.5 cm in size) and IV/II/III (SIV/II/III, 3.5 cm). Therefore, the preoperative diagnosis was DPHC, consistent with HCC in SVIII and iCCA in SIV/II/III. The patient underwent an extended left hemihepatectomy with regional lymph node dissection and cholecystectomy, and DPHC was confirmed histologically.

**CONCLUSIONS:**

Accurate diagnoses and appropriate therapeutic strategies are essential for patients presenting with multiple liver tumors showing heterogeneous imaging features and elevated CA19-9 levels.

## Abbreviations

AFP
: alfa-fetoprotein
CA19-9
: carbohydrate antigen 19-9
DCP
: des-γ-carboxy prothrombin
DPHC
: double primary hepatic cancer
HCC
: hepatocellular carcinoma
HCV
: hepatitis C virus
iCCA
: intrahepatic cholangiocarcinoma
SVR
: sustained virological response

## INTRODUCTION

HCC and iCCA are the most common and second most common primary liver cancers, respectively. In contrast, the synchronous occurrence of these 2 tumor types in different locations of the liver is an uncommon pathology, with a reported occurrence of less than 0.8% of all liver malignancies.^1)^ Here, we report the case of a patient with DPHC treated with surgical resection.

## CASE PRESENTATION

**Fig. 1:**
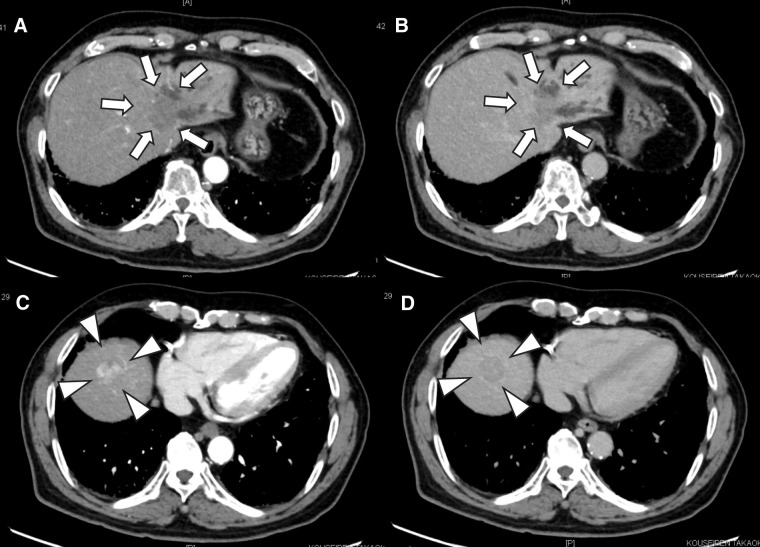
Contrast enhanced dynamic CT images revealing peripheral enhancement with central low density in the arterial phase (**A**) and increased enhancement in the portal venous phase (**B**) at segments IV, II, and III (white arrows). In addition, the tumor at segment VIII is heterogeneously enhanced in the arterial phase (**C**), followed by wash-out in the portal venous phase (**D**) (white arrowheads).

**Fig. 2:**
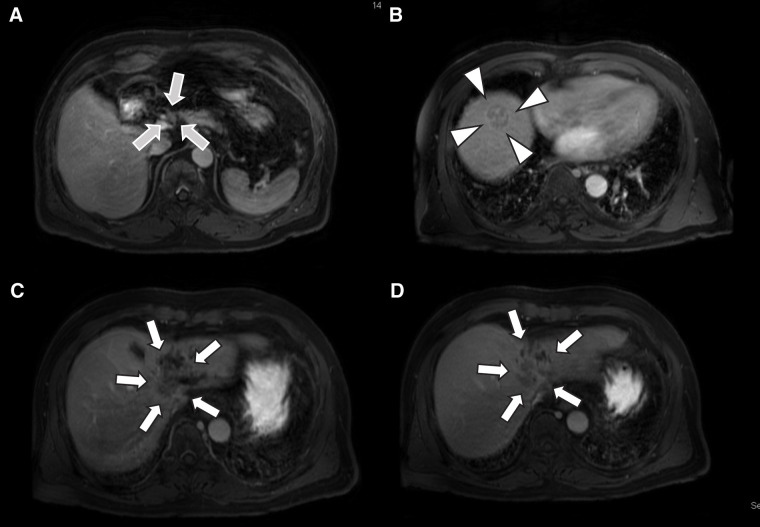
Gadolinium ethoxybenzyl diethylenetriamine pentaacetic acid-enhanced MRI reveals lymph node swelling in the hepatoduodenal ligament (**A**) (gray arrows). Mosaic enhancement of the tumor in segment VIII is also observed (**B**) (white arrowheads). In addition, gradually increasing enhancement, the segment IV, II, and III tumor are detected with slightly unclear margins (**C**, **D**) (white arrows).

**Fig. 3:**
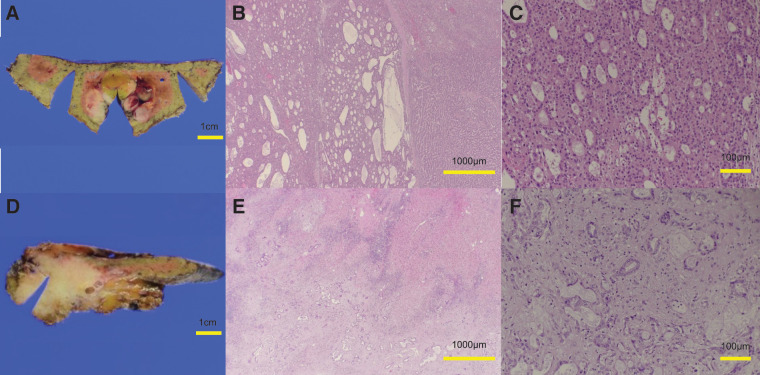
The resected specimen contained a soft, whitish, focally hemorrhagic tumor (size: 3 × 3 cm) in segment VIII (**A**). Histopathological examination revealed that this tumor was a typical, moderately differentiated HCC with a trabecular and pseudoglandular pattern (**B** and **C**). In contrast, a hard, off-white, unclear margin tumor (size: 4 × 3 cm) invaded the adjacent hepatic parenchyma in segments IV, II, and III (**D**). This tumor was pathologically diagnosed as moderately differentiated iCCA with mucin production (**E** and **F**). Scale bars: 1 cm (**A** and **D**), 1000 μm (**B** and **E**), and 100 μm (**C** and **F**). HCC, hepatocellular carcinoma; iCCA, intrahepatic cholangiocarcinoma

**Fig. 4:**
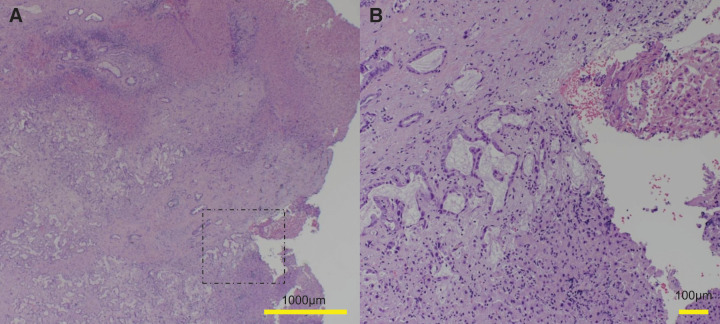
Histopathological examination of the observed cutting edge of the tumors near segment IV, II, and III suggested that a margin-free resection was not achieved (**A**). High magnification view of the area enclosed by the dashed line (**B**) (scale bar in **A**: 1000 μm; scale bar in **B**: 100 μm).

A man in his 70s, suspected of having multiple liver tumors, was referred to us by a pulmonologist. The patient had received medication for idiopathic interstitial pneumonia and ischemic heart disease. He reported a history of smoking and habitual drinking. He had also been treated with a direct-acting antiviral agent for hepatitis C approximately 7 years prior and achieved a SVR. Following SVR, he underwent follow-up every 6 months with regular imaging studies and serological viral marker testing, with no significant findings, except for alcoholic chronic hepatitis. Laboratory results were as follows: platelet count, 23.2 × 10^4^/μL; prothrombin time, 11.4 s (96%); albumin, 4.1 g/dL; and total bilirubin, 0.7 mg/dL. Alanine aminotransferase and aspartate aminotransferase levels were within normal ranges. Hepatic reserve was preserved according to the indocyanine green test (clearance rate = 0.127). The carcinoembryonic antigen, CA19-9, AFP, and DCP levels were 5.2 ng/mL, 36058 U/mL, 3.7 ng/mL, and 159 mAU/mL, respectively. Preoperative abdominal contrast-enhanced CT provided images of 2 separate lesions with different contrast features in segments VIII (SVIII, 2.5 cm in size) and IV/II/III (SIV/II/III, 3.5 cm), suggesting tumors of different origins. On contrast-enhanced dynamic CT, the SIV/II/III tumor showed peripheral enhancement with low central density in the arterial phase, which increased over time in the portal venous and delayed phases (**Fig. 1A** and **1B**). The SVIII tumor was heterogeneously enhanced in the arterial phase, followed by wash-out in the portal venous and delayed phases (**Fig. 1C** and **1D**). In addition, lymph node swelling, suggesting lymph node metastasis, was noted on gadolinium ethoxybenzyl diethylenetriamine pentaacetic acid-enhanced MRI (**Fig. 2A**). This imaging also revealed mosaic enhancement of the SVIII tumor and gradually increasing enhancement of the SIV/II/III tumor, with slightly unclear margins (**Fig. 2B**–**2D**). No abnormal findings were detected on upper or lower gastrointestinal endoscopy. Based on these findings, the patient was diagnosed with DPHC, consistent with the HCC (T2N0M0 Stage II) and iCCA (T3N1M0 Stage IVA) classifications in the 6th edition of the Liver Cancer Study Group of Japan. The patient underwent an extended left hemihepatectomy with regional lymph node dissection and cholecystectomy. The resected specimen contained a soft, whitish, focally hemorrhagic tumor (3 × 3 cm in size) in SVIII and a hard, off-white tumor with an ill-defined margin (4 × 3 cm in size) invading the adjacent hepatic parenchyma in SIV/II/III (**Fig. 3A** and **3D**). The SVIII tumor was histologically confirmed as a typical moderately differentiated HCC with trabecular and pseudoglandular patterns (**Fig. 3B** and **3C**). The SIV/II//III tumor was pathologically diagnosed as a moderately differentiated iCCA with mucin production (**Fig. 3E** and **3F**). In addition, an SIV/II/III tumor was suggested, which could not achieve margin-free resection at the liver cutting edge near the root of the left and middle hepatic veins (**Fig. 4**). Metastases were not observed in the harvested lymph nodes. The hepatic tissue adjacent to the tumor showed no liver cirrhosis. Therefore, based on the histological findings, the liver tumors in this patient were confirmed to be DPHC. The postoperative course was uneventful, and the patient was discharged on POD 13. Postoperative chemotherapy was initiated but discontinued because of worsening of the patient’s chronic lung disease and the patient’s preference. However, the patient died of an acute exacerbation of interstitial pneumonia 7 months postoperatively, with no evidence of tumor recurrence.

## DISCUSSION

HCC and iCCA are the first and second most common types of primary liver cancers, respectively. In contrast, DPHC, defined as the synchronous occurrence of HCC and iCCA in different locations within the same liver, is uncommon, with a reported incidence of less than 0.8% of all primary liver malignancies.^1)^ However, an increasing number of cases have been reported, suggesting that DPHC should be recognized as a distinct clinical entity rather than a purely anecdotal condition. A previous report classified combined HCC and iCCA into 3 types: type A (HCC and iCCA grow independently in different parts of the same liver but have clear boundaries), type B (HCC and iCCA originate from different cells and integrate as they grow; however, there are still certain boundaries between the 2 populations), and type C (HCC and iCCA are completely integrated within the same tumor).^2)^ Goodman et al. proposed another classification system: type I (collision type), type II (transitional type), and type III (fibrolamellar type).^3)^ Based on these classifications, we defined our patient as having either type A or type I.

Previous studies have reported a relationship between chronic liver inflammation and DPHC, mainly due to viral hepatitis, with HCV infection showing one of the strongest correlations.^4,5)^ Watanabe et al. reported a positivity rate of 72.7% for HCV and 9.3% for hepatitis B virus infections in 33 cases of DPHC.^5)^ In the present case, the patient had a history of HCV infection and achieved SVR following direct-acting antiviral therapy. The risk of iCCA development after SVR remains unclear, but residual chronic inflammation may create a carcinogenic substrate for iCCA and HCC, potentially contributing to the onset of DPCH.^6)^ In addition, alcohol-related liver injury was present. Although viral hepatitis has been frequently reported as a background factor in DPHC, the combined contribution of HCV infection and alcohol-related liver injury remains insufficiently characterized, warranting further investigation.

Tumor markers provide important information for the diagnosis of malignant diseases. AFP and DCP are considered the most significant tumor markers for HCC, whereas CA19-9 is a valuable marker for differentiating iCCA from HCC.^7,8)^ A previous study reported that the simultaneous elevation of AFP and CA19-9 in DPHC was significantly higher than in HCC or iCCA alone (29% vs. 9% and 6%).^9)^ In contrast, we observed a simultaneous elevation of DCP and CA19-9 levels in this case. The elevation of DCP might have been due to the patient’s habitual alcohol consumption and related nutritional disorders, including vitamin K deficiency.^10)^ However, the characteristic imaging findings of both tumors made the diagnosis relatively straightforward.

According to the preoperative diagnosis, early accurate clinical diagnosis of DPHC is challenging in many cases.^5)^ Preoperative identification of DPHC remains difficult in routine clinical practice. On contrast-enhanced CT or MRI, the dominant lesion typically demonstrates a “fast wash-in and wash-out” pattern, characteristic of HCC. Additional lesions are therefore often interpreted as satellite nodules or intrahepatic metastases of HCC rather than as independent primary tumors. Consequently, such cases are frequently categorized as “atypical liver cancer,” making accurate preoperative identification of DPHC difficult. In the present case, however, the iCCA was larger than the HCC and demonstrated typical radiological findings, which facilitated differentiation of the 2 lesions as independent primary malignancies.

Importantly, accurate preoperative recognition of DPHC influenced clinical decision-making. If the tumors had been interpreted as HCC with intrahepatic metastasis or satellite nodules, the treatment strategy would likely have consisted of hepatic resection without lymph node dissection or consideration of systemic chemotherapy. Conversely, if both tumors had been diagnosed as iCCA, the disease would have been considered more advanced, and systemic chemotherapy might have been selected as the primary treatment option. Although the diagnosis was DPHC, surgical planning was based on the treatment strategy for iCCA, with the addition of perihilar lymph node dissection to the operative procedure.

Although the prognosis of DPHC is variable, it is generally considered poorer than that of either HCC or iCCA alone, largely depending on the iCCA component. Prognostic outcomes are thought to be primarily determined by factors associated with the iCCA component. There are no definitive findings regarding treatment for DPHC; however, surgical resection is considered curative when feasible. However, careful consideration is warranted when planning surgical intervention in advanced cases like the present case. Indications for surgical resection of iCCA with lesional lymph node metastases are currently being discussed, and no conclusion has been reached, even with the recent advances in liver surgery. Positive lymph node metastases are recognized as an adverse prognostic factor in resected cases.^11,12)^ Some studies have reported the effectiveness of neoadjuvant treatment for advanced iCCA, owing to recent advances in systemic and multidisciplinary treatments.^13)^ However, to the best of our knowledge, there is no evidence regarding the effectiveness of chemotherapy regimens for HCC and iCCA. In our case, the patient and his family strongly desired surgical treatment, despite prudent informed consent. Thus, surgery with perihilar lymph node dissection was chosen as the first treatment method in this case.

This study has some limitations. First, we could not achieve a margin-free resection of the iCCA around the liver cutting edge. Margin-free resection is reportedly a prognostic factor for surgically treated iCCA.^14)^ Moreover, recent advances in the surgical management of iCCA and margin width have been reported as important prognostic factors.^15)^ The patient died of respiratory failure with no evidence of tumor recurrence. Thus, we could not determine the impact of margin status in this case. The natural history of DPHC remains incompletely understood, partly because of its low incidence and heterogeneous clinical presentations. Second, from an oncological perspective, this patient would have been a candidate for adjuvant chemotherapy to improve prognosis; however, treatment was discontinued because of adverse events and the patient’s preference. The patient’s postoperative course was determined not only by the oncological status but also by comorbidities. Various comorbidities should be evaluated cautiously when introducing therapeutic modalities.

## CONCLUSIONS

In summary, we report a case of DPHC in which a preoperative diagnosis was achieved. When multiple liver tumors with discordant imaging characteristics and elevated CA19-9 levels are observed, the possibility of DPHC should be considered to facilitate appropriate diagnostic evaluation and therapeutic planning.

## DECLARATIONS

### Availability of data and materials

All data generated or analyzed during this study are included in the published article.
